# Incidence and case fatality rates of community-acquired pneumonia and pneumococcal diseases among Korean adults: Catchment population-based analysis

**DOI:** 10.1371/journal.pone.0194598

**Published:** 2018-03-29

**Authors:** Jung Yeon Heo, Yu Bin Seo, Won Suk Choi, Jacob Lee, Jin Gu Yoon, Saem Na Lee, Min Joo Choi, Ji Yun Noh, Jin-Young Ahn, Hye Won Jeong, Hee Jin Cheong, Woo Joo Kim, Hee Young Lee, Joon Young Song

**Affiliations:** 1 Department of Infectious Diseases, Ajou University School of Medicine, Suwon, Republic of Korea; 2 Division of Infectious Diseases, Department of Internal Medicine, Hallym University College of Medicine, Chuncheon, Republic of Korea; 3 Division of Infectious Diseases, Department of Internal Medicine, Korea University College of Medicine, Seoul, Republic of Korea; 4 Asian Pacific Influenza Institute (APII), Korea University Guro Hospital, Seoul, Korea; 5 Department of Internal Medicine, Chungbuk National University College of Medicine, Cheongju, Republic of Korea; 6 Division of Pulmonary and Critical Care Medicine, Department of Internal Medicine, Chungbuk National University Hospital, Cheongju, Republic of Korea; 7 Division of Infectious Diseases, Department of Internal Medicine, Chungbuk National University Hospital, Cheongju, Republic of Korea; 8 Center for Preventive Medicine and Public Health, Seoul National University Bundang Hospital, Seongnam, Republic of Korea; Public Health England, UNITED KINGDOM

## Abstract

**Background:**

Pneumonia is a leading infectious cause of morbidity and mortality among adults. Pneumococcal pneumonia (PP) is the most common vaccine-preventable bacterial etiology of pneumonia. In this study, we estimated the incidence of community-acquired pneumonia (CAP) and pneumococcal diseases among Korean adults.

**Methods:**

Clinical and microbiological databases from three hospitals were retrospectively reviewed to determine the incidence and case fatality rates of CAP and pneumococcal diseases in Korean adults aged ≥19 years from 2011 to 2014. Incidence and case fatality rates of CAP, PP and invasive pneumococcal diseases (IPD) were evaluated based on the catchment population. Catchment population was calculated using national health insurance data, estimating the proportion of patients with pneumonia that were medically attended at each hospital.

**Results:**

Among 5,783 patients with medically attended CAP, 833 (14.4%) had PP. For IPD, a total of 91 culture-confirmed cases were identified. The overall incidence of CAP was 307.7 cases per 100,000 persons per year with an in-hospital mortality rate of 6.2%. The estimated annual incidence of pneumococcal pneumonia was 42.2–49.4 cases per 100,000 persons per year, increasing with age to >280 per 100,000 persons per year in older patients over 70 years. The annual incidence of IPD had a range of 4.1–6.5 cases per 100,000 persons per year. The overall case fatality rate for invasive pneumococcal diseases was 30.8% with the highest rate of 66.7% in patients over 80 years.

**Conclusion:**

Over the study period, incidences of CAP, PP and IPD were consistently high, particularly in older people. These results provide baseline data to establish healthcare strategies and estimate their impact among Korean adults.

## Introduction

Pneumonia is a leading cause of morbidity and mortality worldwide, with 450 million annual cases with 1.3 million annual deaths in children and 1.6 million in adults aged ≥60 years [1–4]. *Streptococcus pneumoniae* (pneumococcus) is the most common bacterial pathogen causing community-acquired pneumonia (CAP). However, the reported burden of pneumonia and pneumococcal diseases varies temporally and geographically [5,6]. Disease burden might increase or decrease with changes in climate, population density or distribution, social activity and vaccination rates. In all countries, characterization of pneumonia and pneumococcal disease burden is crucial for establishing public health policy, including national immunization programs.

Annual incidence of CAP is reported to be 520–610 cases per 100,000 adults, increasing in older people [5]. Among CAP cases, 24.0–27.5% are attributable to pneumococcus, according to meta-analyses [7,8]. In particular, 5–10% of pneumococcal pneumonia is bacteremic, which accounts for 9–18 cases per 100,000 adults annually [9–12]. The incidence of pneumococcal pneumonia (PP) and invasive pneumococcal disease (IPD) is expected to rise with an increasingly older population, although widespread use of pneumococcal conjugate vaccine (PCV) may reduce pneumococcal disease burden. After the introduction of PCV13, many countries carried out cost-effectiveness analyses for pneumococcal vaccination strategies, comparing PCV13 to the 23-valent pneumococcal polysaccharide vaccine (PPSV23) [13–17]. Most studies have suggested that PP incidence might be an important factor for determining the cost effectiveness of pneumococcal vaccination [13–16].

In South Korea, the National Immunization Program (NIP) provides free vaccination with PPSV23 for old adults; NIP was implemented for everyone aged ≥65 years in May 2013. Since May 2014, pediatric PCV10/PCV13 vaccination is included in the NIP, so all children can receive pneumococcal vaccinations. However, the burden of PP and IPD among adults is not well understood, impeding selection of optimal strategies and cost-benefit analyses for pneumococcal vaccination in South Korea.

This study was planned to better understand the burden of pneumonia and pneumococcal diseases among adults in South Korea. We estimated the population-based incidence and case fatality rates of CAP, PP and IPD using a hospital catchment population.

## Methods

### Study design

This retrospective study was conducted at three university hospitals (Korea University Guro Hospital, Hallym University Kangnam Sacred Heart Hospital and Chungbuk National University Hospital) in different areas of South Korea between January 1, 2011 and December 31, 2014. This study was designed to determine the annual incidence and case fatality rates of CAP, PP and IPD, stratified by age group. To estimate incidences, numerators were defined as number of eligible cases (inpatient and outpatient) with CAP, PP and IPD in the study hospitals. Hospital catchment population was the denominator, calculated using the National Health Insurance Sharing Service (NHISS) database.

The study was performed with approval of the institutional review boards at Korea University Guro Hospital, Hallym University Kangnam Sacred Heart Hospital and Chungbuk National University Hospital. Written informed consent was waived because all data were collected retrospectively by reviewing medical records. In addition, all medical records were fully anonymized before we accessed them.

### Calculation of pneumonia catchment population

For each study year, the pneumonia catchment population of the university hospitals was calculated using data from the hospital and the NHISS database (S1 Table). Calculations used the following formula:

Pneumonia catchment population of each hospital = Σ_*i*_ Σ_*j*_ Σ_*k*_ *A*_*ijk*_

$$A_{ijk}=\frac{H_{ijk}}{M_{ijk}}\times P_{ijk}$$

*i* = *age*, *j* = *gender*, *k* = *area*

*Aijk* = *pneumonia catchment population of a particular* gender/age *group in a particular area*

*Hijk* = *number of pneumonia patients in a particular area with a particular gender and age who visited the hospital*

*Mijk* = *number of pneumonia patients in a particular area with a particular gender and age who visited any medical institutions*

*Pijk* = *number in the population in a particular* gender/age *group in a particular area*

We defined the catchment area of a study hospital as the collection of administrative divisions that had a visit from one or more patients with pneumonia. In South Korea, administrative divisions are composed of 262 areas that include 75 cities, 86 counties and 101 districts across the country. In each regional area, the proportion of patients with pneumonia visiting each study hospital was estimated by age group and gender using the NHISS database. The number of patients with pneumonia registered in catchment areas of the three study hospitals was 33,353 in 2011, 42,519 in 2012, 38,957 in 2013 and 28,778 in 2014. The number of hospitals that provided medical services to the patients was 2,718 in 2011, 2,818 in 2012, 2,693 in 2013 and 2,850 in 2014. For each area, the proportion was multiplied by the number in the population by age group and gender. Using this process, age group-specific and gender-specific catchment populations were estimated for each area, and the sum of these estimates was used as the catchment population for each of the hospitals. During the study period from 2011 to 2014, the catchment population was estimated annually (S1 Table).

### Estimation of disease incidence and case fatality rates

To estimate disease incidence of CAP, PP and IPD, annual numbers of cases were estimated by reviewing clinical and microbiological databases during the study period from January 1, 2012 through December 31, 2014. For each hospital, two infectious disease specialists reviewed all cases of pneumonia (ICD-10 codes J10–18 and J69) and selected those meeting the CAP criteria [12]. Among cases, those of PP were further classified based on the diagnostic definition [12]. For IPD, all cases with pneumococcal isolates were reviewed for clinical findings and specimen collection sites. Information on age, gender, diagnosis (CAP, PP and IPD) and in-hospital mortality were obtained. Disease incidences were estimated using the pneumonia catchment population as a denominator. Patients were stratified by age group (19–49 years, 50–69 years, 70–79 years, and ≥80 years). Case fatality rates (in-hospital mortality) were estimated as the proportion of deaths among designated cases (people with CAP, PP or IPD).

### Case definitions

CAP was defined based on clinical and radiological criteria of: (a) acute pulmonary infiltrate evident on chest radiographs and consistent with pneumonia within 48 h after admission, (b) confirmatory findings on clinical examination, and (c) acquisition of the infection outside a hospital [18]. For patients with CAP, PP was determined if blood samples or adequate lower respiratory specimens yielded pneumococcal isolates that were optochin sensitive and had alpha hemolytic colonies in the clinical laboratory [12]. Patients with positive Binax NOW *Streptococcus pneumoniae* urinary assay were also diagnosed as having PP [15]. Adequate lower respiratory specimens included transbronchial aspirates, broncho-alveolar lavage (BAL) specimens, and sputum specimens with predominant presence of gram-positive diplococci on high-quality Gram staining of (>25 WBCs and <10 squamous epithelial cells/low-power field). IPD was defined as an infection confirmed by isolation of *S*. *pneumoniae* from a normally sterile site such as blood, cerebrospinal fluid, or pleural or ascitic fluid [19].

### Data analysis

Incidences were calculated as proportions by dividing the number of patients (numerator) with the catchment population (denominator), and expressed as number of cases per 100,000 persons per year with 95% confidence intervals. Age-dependent and year-dependent trends for disease incidence and case-fatality rate were analyzed by linear-by-linear chi-square test. SPSS version 20.0 (SPSS for Windows, SPSS, Chicago, IL, USA) was used for statistical analysis. *P* values < 0.05 were considered statistically significant.

## Results

### Annual incidence and mortality: Community-acquired pneumonia, pneumococcal pneumonia and invasive pneumococcal diseases

During periods from 2011 to 2014, the study hospital catchment areas covered 193 areas of administrative divisions across the country. The number of patients in the pneumonia catchment population in the study hospitals was 1,871,312 (S1 Table). The proportion of patients with pneumonia visiting the study hospitals was 3.6% of all pneumonia patients in the hospital catchment area. A total of 5,783 patients with CAP aged ≥19 years were verified, and 833 (14.4%) among those had PP. For IPD, 91 cases were identified. The estimated annual incidence of CAP was 307.7 patients (95% confidence interval [CI], 307.0 to 308.4) per 100,000 persons with an overall in-hospital mortality of 6.2% (Table 1). Estimated annual incidences were 44.3 (95% CI, 44.3 to 44.4) for PP and 4.8 (95% CI, 4.8 to 4.9) for IPD, both per 100,000 persons. Overall case-fatality rates were 9.1% (95% CI 7.4 to 11.3) for PP and 30.8% (95% CI 22.2 to 40.9) for IPD. Although incidence varied annually, CAP incidence tended to increase from 2011 to 2014. The annual incidences of PP and IPD were not significantly changed.

**Table 1:** The estimated annual incidence (per 100,000 persons) and case-fatality rate of invasive pneumococcal disease (IPD), pneumococcal pneumonia (PP) and community-acquired pneumonia (CAP).

Incidence rate (95% confidence interval)						
	Total	2011	2012	2013	2014	p-value
IPD	4.8 (4.8–4.9)	6.5 (6.4–6.5)	4.2 (4.2–4.3)	4.1 (4.1–4.2)	4.4 (4.4–4.5)	0.161
PP	44.3 (44.3–44.4)	42.2 (42.0–42.3)	42.6 (42.5–42.8)	43.1 (43.0–43.3)	49.4 (49.2–49.5)	0.104
CAP	307.7 (307.0–308.4)	288.7 (287.4–290.0)	297.8 (296.5–299.1)	336.0 (334.6–337.4)	309.6 (308.3–310.9)	0.005
Case-fatality rate (95% confidence interval)						
IPD	30.8 (22.2–40.9)	28.1 (15.6–45.4)	31.6 (15.4–54.0)	36.8 (19.2–59.0)	28.6 (13.8–50.0)	0.836
PP	9.1 (7.4–11.3)	10.5 (7.1–15.4)	7.8 (4.8–12.5)	9.1 (5.8–13.9)	9.0 (5.9–13.3)	0.698
CAP	6.2 (5.6–6.8)	6.9 (5.7–8.3)	5.7 (4.6–7.1)	5.9 (4.8–7.2)	6.2 (5.1–7.6)	0.524

### Age-stratified incidence and mortality: Community-acquired pneumonia, pneumococcal pneumonia and invasive pneumococcal diseases

Age-stratified incidence and case-fatality rates for CAP significantly increased with age (p < 0.001): the highest incidence was in the age group of ≥80 years with 4,865.7 cases (95% CI, 4,798.0 to 4,934.0) per 100,000 persons per year (Table 2). The age-stratified CAP incidence for the age group 70 years or older was 12.4-fold higher than under age 70 years (2,236.0 per 100,000 vs. 179.4 per 100,000). The case-fatality rate for CAP was also the highest for the age group of ≥80 years, at 11.4% (Table 2). Age-stratified incidence and case-fatality rates for PP and IPD increased with age (p < 0.001). Age-stratified incidence was highest for the age group of ≥80 years, at 676.1 cases per 100,000 persons (95% CI 669.7 to 682.4) for PP and 57.1 per 100,000 (95% CI 54.0 to 60.3) for IPD. Age-stratified incidences of PP and IPD for people 70 years or older increased more than 15.0-fold (357.1 per 100,000 vs. 23.6 cases per 100,000 persons) for PP and 8.6-fold (28.1 per 100,000 vs. 3.2 per 100,000) for IPD, compared to people under age 70. The proportion of PP among all CAP cases did not change over the study period (p = 0.541), but significantly increased with age (p = 0.001): the highest proportion was 17.1% for the age group of 70–79 years and the lowest was 9.5% for the age group of 19–49 years (Table 3).

**Table 2:** Age-stratified incidence (per 100,000 persons per year) and case-fatality rate of invasive pneumococcal disease (IPD), pneumococcal pneumonia (PP) and community-acquired pneumonia (CAP).

Incidence rate (95% confidence interval)					
	19–49 years	50–69 years	70–79 years	≥80 years	p-value
IPD	1.5 (1.4–1.5)	7.4 (7.4–7.5)	21.9 (21.6–22.1)	57.1 (54.0–60.3)	<0.001
PP	9.1 (9.0–9.1)	56.6 (56.4–56.7)	287.4 (284.5–290.3)	676.1 (669.7–682.4)	<0.001
CAP	95.0 (94.9–95.0)	369.7 (368.4–371.0)	1679.6 (1656.0–1704.0)	4865.7 (4798.0–4934.0)	<0.001
Case-fatality rate (95% confidence interval)					
IPD	11.1 (3.1–32.8)	22.5 (12.3–37.5)	42.9 (24.5–63.5)	66.7 (39.1–86.2)	0.031
PP	3.6 (1.4–9.0)	6.2 (4.0–9.5)	11.6 (8.3–15.9)	14.1 (9.3–20.8)	<0.001
CAP	1.1 (0.7–1.9)	4.5 (3.7–5.5)	8.6 (7.3–10.0)	11.4 (9.6–13.4)	<0.001

**Table 3:** Year- and age-dependent trend analysis—Proportion of pneumococcal pneumonia (PP) among community-acquired pneumonia (CAP).

Year	2011	2012	2013	2014	p-value
Proportion (%, 95% CI)	14.8 (13.0–16.7)	14.3 (12.5–16.3)	12.8 (11.3–14.6)	16.0 (14.2–17.9)	0.541
PP (No.)	209	192	198	234	
CAP (No.)	1431	1342	1543	1467	
Age	19–49 years	50–69 years	70–79 years	≥80 years	p-value
Proportion, (%, 95% CI)	9.5 (8.0–11.4)	15.3 (13.8–17.0)	17.1 (15.4–19.0)	13.9 (11.9–16.2)	0.001
PP (No.)	110	305	276	142	
CAP (No.)	1154	1994	1613	1022	

CI, confidence interval

## Discussion

In this study, the overall estimated incidence of CAP was annually 307.7 cases per 100,000 persons. This number increased with age, up to 4,865.7 cases per 100,000 people aged 80 years and older. Overall estimated annual incidences were 44.3 cases per 100,000 for PP and 4.8 per 100,000 for IPD, increasing to 676.1 for PP and 57.1 for IPD per 100,000 persons in adults aged ≥80 years. From incidence and population data based on 2015 resident registration for South Korea (41,722,000 adults aged ≥19 years), we calculated around 128,000 cases of CAP, 18,500 cases of PP and 2,000 cases of IPD in Korean adults annually. Although CAP is a relatively frequent infectious disease in developed and developing countries, only a few studies are available on the incidence of CAP [20–23]. The incidence for PP is usually extrapolated from the proportion of *S*. *pneumoniae*-caused CAP cases from reports on CAP etiology. Although a few Korean reports present the characteristics and etiology of CAP, no data provide information on the population-based incidences of CAP, PP or IPD [24–28]. This lack of data is a barrier to deciding public health policy for pneumococcal vaccination strategies.

The most notable findings of this study were the epidemiological trend of the annual incidence of CAP, PP and IPD from 2011 to 2014. Although there was a relatively brief period for understanding the epidemiological trend, CAP incidence annually tended to increase from 2011 to 2014. This trend was also observed in a recent study for hospitalized CAP in South Korea [29]. According to Korean Statistics, the death rate caused by pneumonia was markedly increased from 8.1 per 100,000 persons in 2000 to 23.7 per 100,000 persons in 2014 [30]. The death rate due to pneumonia in adults aged 15–64 years was were not changed from 2.2 per 100,000 persons in 2000 to 2.3 per 100,000 persons in 2014, while it was rapidly increasing in elderly aged ≥65 years from 92.6 per 100,000 persons in 2000 to 177.5 per 100,000 persons in 2014. Therefore, the increasing incidence of CAP over the years may be attributed to increased elderly population. Nonetheless, the annual incidences of PP and IPD were not significantly changed. The free vaccination program using PPSV23 was introduced for all persons aged ≥65 years in South Korea since May 2013. After the introduction of PPSV23 NIP, the PPSV23 coverage rate among the population aged ≥65 years increased sharply: 5.0% in May 2013, 44.5% in December 2013 and 57.3% in December 2014 [31]. Together with this strategy for elderly, private PCV10/PCV13 vaccination has been widely implemented in children since March 2010, reaching about 65% coverage rates in 2012 [32]. Considering the direct and indirect effect by pneumococcal vaccination of old age and childhood, the incidences of adult pneumococcal diseases are expected to decrease gradually over the next few years.

The annual incidence rate of CAP in adults was estimated to vary between 162 and 1001 per 100,000 persons depending on the country: United States (248 cases per 100,000 per year), Germany (370 to 1001 cases per 100,000 per year), Spain (162 cases per 100,000 per year) and Japan (960 per 100,000 person-years) [20–23]. Compared with our estimates, the overall incidence of CAP in South Korea was lower than in Germany and Japan, but higher than in the United States and Spain. This difference might result from differences in enrollment criteria, population structure and healthcare system by country. In addition to these factors, the denominator for estimating incidence rate varies by country. Spain and Japan studies presented the number of CAP cases reported by physicians in the total population in a given area, while German study estimated CAP incidence based on a covered population by community-based hospitals participating in the surveillance network [20,22,23]. Thus, basic differences need to be considered when comparing incidences between studies. In the United States, the incidence of CAP requiring hospitalization was calculated for the adjusted population of a hospital catchment area [21]. In this study, the hospital-based pneumonia catchment population was used as a covered population similar to in German and American studies [20,21]. In South Korea, CAP and PP are not national notifiable infectious diseases requiring obligatory report. Estimating the incidence of infectious diseases such as pneumonia and pneumococcal diseases is difficult because of an easily accessible healthcare system even to hospitals in distant areas. Korean patients are free to choose any medical facilities or physicians across the country. They can go directly to specialized physicians in secondary/tertiary hospitals, bypassing general practitioners. Thus, evaluating the incidence in a confined area would not be feasible. The proportion of people covered by the National Health Insurance Program is over 98% of South Korean population [33]. We could calculate a hospital catchment population as potential hospital users by analyzing health care utilization of patients diagnosed with pneumonia from the NHISS database. This method enabled us to estimate the incidence of PP, IPD and CAP based on a hospital database.

We documented the annual incidences of PP (44.3 cases per 100,000) and IPD (4.8 cases per 100,000) among adults and their increase with age. PP comprised 14.4% of all-cause CAPs. Although Song et al. previously reported an increased clinical and economic burden of IPD by age and risk groups in South Korea, they did not reported the IPD incidence because of study limitations [26]. Estimating the burden of non-bacteremic PP is more problematic than estimating IPD. Sputum cultures for identifying PP show limited specificity [12]. Prescription of empirical antibiotics before microbiologic tests increase the proportion of CAPs with unknown etiology. However, the use of a pneumococcal urine antigen test (UAT) contributes to diagnosing pneumococcal pneumonia [7]. The reported proportion of pneumococcal pneumonia is 7.4–13.5% of all-cause CAP cases in South Korea [25,28]. Yoo et al. found a PP rate of 7.4% in patients aged ≥50 years with CAP [28]. This proportion is relatively lower than proportions from meta-analyses. However, our study showed that the proportion of PP among all-cause CAP was 14.4% and significantly increased with increasing age when UAT was applied to diagnose PP in addition to sputum cultures. The incidence of IPD among US adults was estimated to be annually 14.0–58.0 per 100,000 persons in the early 2000s and 11.0–16.2 per 100,000 persons in Scotland and Germany at around the same period [34–36]. In Japan, a relatively low incidence of 1.8–4.4 per 100,000 people was seen among people aged ≥65 years [13]. Given that the incidence of IPD was annually 10.9 cases per 100,000 adults aged ≥50 years in our study, the incidence of IPD in South Korea was lower than those in Europe and the United States, but higher than that of Japan. Prior antibiotic use might reduce the sensitivity of blood culture. The frequency of positive blood cultures might be low because of autolysin release from pneumococci during stationary growth phase, resulting in an underestimation of IPD [12,27]. In addition, the indirect effect of childhood pneumococcal vaccination, which had about 65% coverage using a 10/13-valent pneumococcal conjugate vaccine as of 2012, might affect IPD incidence [37]. Of note, the incidence of CAP and IPD varies by countries but case-fatality rates in the present study were similar to those of previous reports: 5 to 14% for CAP and 15 to 30% for IPD [8,19,22,24,26,38].

The primary strength of this study was that we identified CAP, PP and IPD cases through a reliable review process by infectious disease experts. We estimated the incidence and case-fatality rate longitudinally over four-year study periods. Contrary to studies using the NHISS database alone, we avoided overestimation by excluding healthcare-associated pneumonia cases by reviewing medical records. In the recent study in South Korea, the annual CAP incidence was estimated to be 626 per 100,000 persons, which was higher than that of the present study [29]. In addition the study periods, such a difference in the study methods might affect the results. On the other hand, the study had some limitations. First, it might not represent the nationwide incidence of CAP. Incidence of CAP throughout South Korea may vary according to geographic areas, healthcare accessibility, and population characteristics. Second, we could not assess indirect effects from childhood PCV10/PCV13 immunization thoroughly because this study was conducted soon after the introduction of a national immunization program. Third, this study was retrospectively conducted. The retrospective nature of data may underestimate the incidence of pneumococcal diseases, considering that the diagnostic tests of these diseases was carried out by physician’s decision. Good performance of diagnostic tests (sputum culture, urinary antigen test and blood culture) would be crucial for the diagnosis of pneumococcal diseases, but these might be conducted irregularly.

In conclusion, the incidences of CAP, PP and IPD were consistently high, particularly in older patients over the study period. The proportion of PP increased markedly with age. These results provide baseline data to establish healthcare strategies and estimate their impact among Korean adults.

## Supporting information

S1 Table Age-stratified catchment population from 2011 to 2014. (DOCX)
