# Supplementary material for: Incidence and case fatality rates of community-acquired pneumonia and pneumococcal diseases among Korean adults: Catchment population-based analysis
Source: PLoS One. 2018 Mar 29;13(3):e0194598. doi: 10.1371/journal.pone.0194598 (PMC5875769; doi:10.1371/journal.pone.0194598)
Supplement: S1 Table — (DOCX) [file pone.0194598.s001.docx]

**S1 Table. Age-stratified catchment population from 2011 to 2014**

| Estimated catchment populations (No.) | | | | | | |
| --- | --- | --- | --- | --- | --- | --- |
| Age group |  | 19-49 years | 50-69 years | 70-79 years | ≥80 years | Total |
| Year | 2011 | 334,574 | 133,668 | 21,457 | 3957 | 493,656 |
|  | 2012 | 302,274 | 119,545 | 20,025 | 6739 | 448,583 |
|  | 2013 | 280,693 | 145,125 | 26,110 | 5272 | 457,200 |
|  | 2014 | 297,370 | 141,024 | 28,443 | 5036 | 471,873 |
| Total |  | 1,214,911 | 539,362 | 96,035 | 21,004 | 1,871,312 |
